# Growth efficiency, intestinal biology, and nutrient utilization and requirements of black soldier fly (*Hermetia illucens*) larvae compared to monogastric livestock species: a review

**DOI:** 10.1186/s40104-022-00682-7

**Published:** 2022-05-05

**Authors:** Mohammad M. Seyedalmoosavi, Manfred Mielenz, Teun Veldkamp, Gürbüz Daş, Cornelia C. Metges

**Affiliations:** 1 Research Institute for Farm Animal Biology (FBN), Institute of Nutritional Physiology, 18196 Dummerstorf, Germany; 2 Wageningen UR, Livestock Research, P.O. Box 338, 6700AH Wageningen, Netherlands

**Keywords:** Black soldier fly, Comparative growth performance, Farm animals, Insects as feed, Intestinal function, Nutrient cycles, Nutrition and physiology

## Abstract

In recent years, interest in the larvae of black soldier fly (BSF) (*Hermetia illucens*) as a sustainable protein resource for livestock feed has increased considerably. However, knowledge on the nutritional and physiological aspects of this insect, especially compared to other conventional farmed animals is scarce. This review presents a critical comparison of data on the growth potential and efficiency of the BSF larvae (BSFL) compared to conventional monogastric livestock species. Advantages of BSFL over other monogastric livestock species includes their high growth rate and their ability to convert low-grade organic waste into high-quality protein and fat-rich biomass suitable for use in animal feed. Calculations using literature data suggest that BSFL are more efficient than broilers, pigs and fish in terms of conversion of substrate protein into body mass, but less efficient than broilers and fish in utilization of substrate gross energy to gain body mass. BSFL growth efficiency varies greatly depending on the nutrient quality of their dietary substrates. This might be associated with the function of their gastrointestinal tract, including the activity of digestive enzymes, the substrate particle characteristics, and their intestinal microbial community. The conceived advantage of BSFL having an environmental footprint better than conventional livestock is only true if BSFL is produced on low-grade organic waste and its protein would directly be used for human consumption. Therefore, their potential role as a new species to better close nutrient cycles in agro-ecological systems needs to be reconsidered, and we conclude that BSFL is a complementary livestock species efficiently utilizing organic waste that cannot be utilized by other livestock. In addition, we provide comparative insight into morpho-functional aspects of the gut, characterization of digestive enzymes, gut microbiota and fiber digestion. Finally, current knowledge on the nutritional utilization and requirements of BSFL in terms of macro- and micro-nutrients is reviewed and found to be rather limited. In addition, the research methods to determine nutritional requirements of conventional livestock are not applicable for BSFL. Thus, there is a great need for research on the nutrient requirements of BSFL.

**Supplementary Information:**

The online version contains supplementary material available at 10.1186/s40104-022-00682-7.

## Introduction

According to the UN population projections, the world’s population is expected to reach 9.6 billion by 2050 [1] and FAO estimates that food production will need to increase by 70% to feed this larger and higher-income global population [2]. Most concerning is the increasing demand for meat, milk and eggs at the expense of staple foods, putting further pressure on already scarce agricultural resources, taking up additional land with negative impacts on water, soil, and air quality, and leading to increased greenhouse emissions and reduced biodiversity [3]. Increasing food production without expanding land use is therefore of paramount importance, and knowledge of cost-effective and sustainable feed alternatives in circular agricultural systems can improve sustainability [4].

Over the past two decades, there has been a surge of interest in the study of insects for feed and food as evidenced by the exponential growth in the numbers of publications (693%) and citations (71,477%) using the term ‘insect farming’ [5], for example, when compared to the increase in the numbers of publications (147%) and citations (446%) using the search term ‘chicken’ over the same period. Insects are a promising source of high quality protein, fats, and certain minerals, can be farmed in high densities and have a high bioconversion ratio [6]. In addition, many insect species can be grown on organic biomass waste. This helps recycle nutrients from the environment that otherwise become a source of air, soil and water pollution, and have negative impacts on biodiversity and climate [7, 8]. In this regard, there is great interest in the larvae of the saprophytic black soldier fly (BSF) (*Hermetia illucens*) which can contribute to better management of organic and inorganic nutrient resources, in particular recycling of nitrogen and phosphorus.

**Fig. 1:**
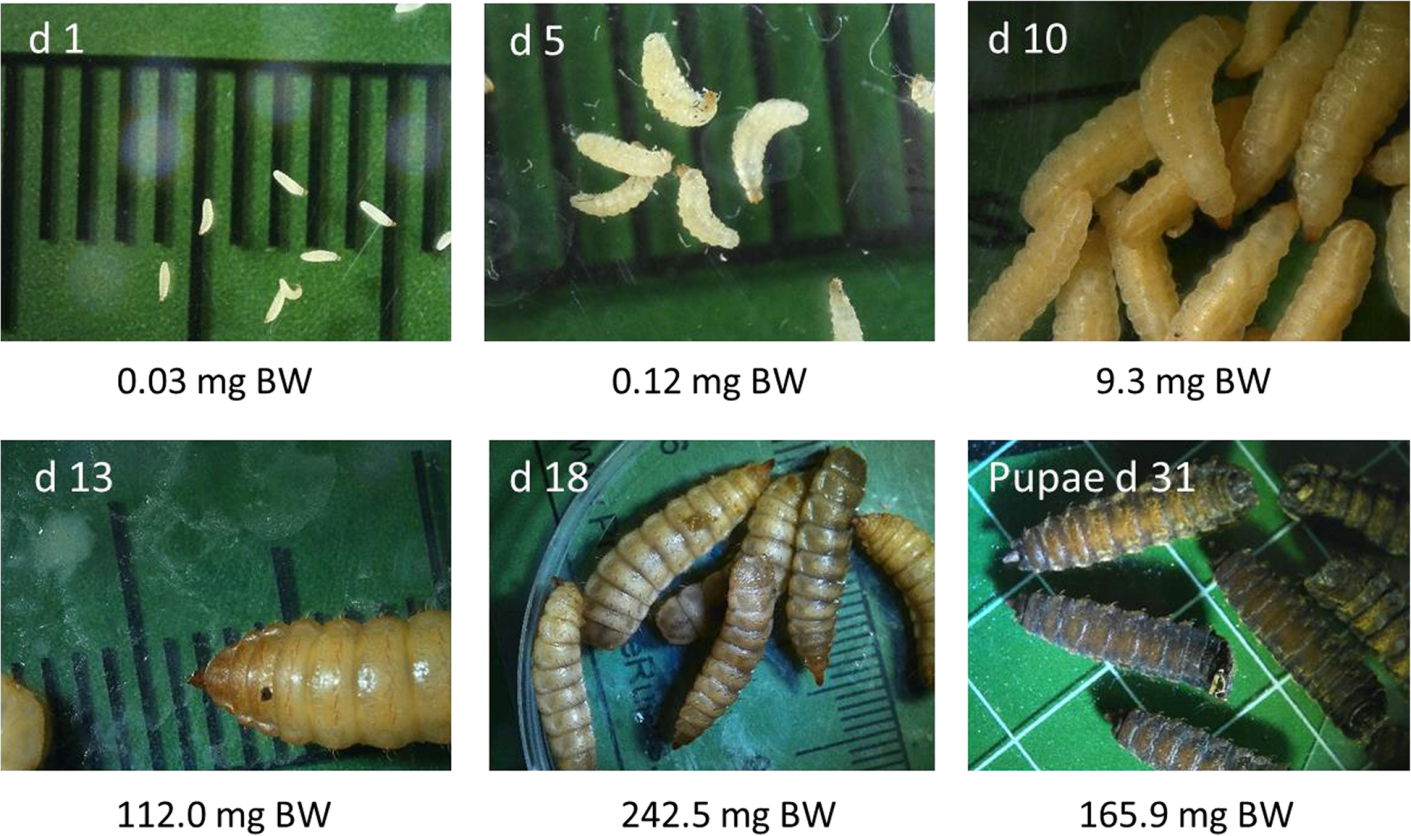
Development of black soldier fly larvae from d 1 after hatching up to the pupal stage. The photographs are showing the development of larvae which were reared on chicken feed together with the corresponding mean body weight (wet weight) of at least 100 larvae; the body weight development corresponds to an 8083 fold increase from d 1 to d 18 (maximum body weight at d 18); each division of the scaling from d 1 to d 18 represents 1 mm; at d 31 the division of the scaling represents 10 mm

The BSF is a wasp-like fly, originally native to the Americas, widespread in tropical and temperate regions of the world [9], and currently being domesticated worldwide [10]. The species is characterized by a short life cycle of approximately 41 d, which can increase up to 131 d depending on the nutrient and energy composition of feeding substrates and ambient temperature of rearing environment [11]. The short life cycle coupled with a fast growth potential of BSF larvae (BSFL) (Fig. 1) make this species an interesting one suitable for farming conditions.

Compared to other farmed insect species such as the larvae of mealworm, BSFL is known to feed and develop on a wider range of feed sources such as kitchen waste [12, 13], manure [14–16], faecal sludge [17], and distillers’ by-products [18], converting it to high quality protein (41% to 54% of dry matter (DM)) [19, 20]. However, the nutrient requirements of BSFL are insufficiently known which is necessary to design optimized diets and to predict the efficiency of low-quality substrates, and how they can be nutritionally upgraded or combined to achieve high feed efficiency. The protein quality of BSFL is similar to soybean protein but has a higher methionine and tyrosine content [21] and BSFL has been used as a partial replacement of soybean meal and fishmeal in pig [4, 22, 23], broiler [24, 25] and fish diets [26]. Furthermore, BSFL accumulate large amounts of fat (11.8–41.7% of DM) with more saturated fatty acids than other insects [9, 16]. In addition, BSFL meal might be an interesting source of calcium, phosphorus and other minerals [18, 27]. Recently, the European Commission authorized the use of processed animal protein derived from insects for feeding aquaculture animals, pigs and poultry [28]. For the suitability of BSFL as a component in diets for swine, poultry and fish species we refer the reader to previous reviews [29–34].

Given that BSFL are currently reared to produce feed and not food, a one-to-one comparison of BSFL with conventional farmed animal species, particularly for resource utilization efficiency and environmental impact involves pitfalls. We propose that the larvae of *Hermetia illucens* are not in direct competition with conventional farmed animal species, but rather constitute a complementary species that can efficiently utilize low-grade feedstuff and organic waste, which otherwise cannot be utilized by other livestock. Only such an approach enables the integration of BSFL into agro-ecological systems to close nutrient cycles as efficiently as possible. The objective of this paper is to present the current knowledge on growth potential, feed efficiency, respiratory gas exchange, and intestinal biology of BSFL with emphasis on enzymatic and microbial digestion compared to conventional monogastric livestock species. These comparisons examine potential physiological differences between BSFL and conventional monogastric livestock species, to identify knowledge gaps, and do not necessarily imply that one species should be favored over another in commercial farming. Based on the recognition that methodology used for conventional farmed animals to estimate nutritional requirements is less suitable for BSFL, we also provide an overview of the available data on nutrient utilization and requirements (macronutrients and minerals) for BSFL. Finally, we present some opportunities for future research in BSFL nutritional characteristics and requirements.

## Feeding behavior and substrate scale

A unique feature of BSFL is the rapid feeding rate, for which there are specific behavioral mechanisms. For example, a group of larvae moves in a coordinated manner to form fountains around the feed, into which new larvae crawl from below and are pumped out at the top, making the feed accessible to all larvae in the group in a time-efficient way [35]. This behavior is particularly important when the amount of available feed is limited and is supported by their comparatively hard structure of the mouth apparatus containing 0.49% calcium [35] which has been referred to as a “tunnel boring machine” [36] that aids in exploration and crushing of the feeding substrate. Under farming conditions, where feed is abundant, regulation of temperature and thus metabolism by body activity could be an equally important behavior [37]. The aggregation behavior of BSFL, termed larval-mass effect, is typical e.g. for necrophagous diptera and helps to increase the body temperature, thereby reducing the developmental time [38]. With their voracious feeding habit, BSFL are omnivorous feeders on a wide range of organic matter of both animal and plant origin [10]. Based on estimates of Shishkov et al. [35] and depending on moisture content of the substrate, BSFL can consume daily 2 to 6.5 times their body mass in feed. One factor determining the BSFL feeding behavior is the physical structure of the feeding substrate. As reviewed by Barragan-Fonseca et al. [39] layer depth (i.e. oxygen availability), density, homogeneity and moisture of feeding substrates affect BSFL performance and survival. Brits [40] suggested that larvae require more energy to consume substrates with large particles than those with small particles (range 1 to 9 mm). Also zones of varying nutrient composition through which larvae need to move lead to lower bioconversion [40]. Liland et al. [41] discussed a particle size of about 0.15 mm that might be preferred by larvae for ingestion. However, detailed information on the physical structure of the feeding substrate of BSFL is missing.

It was previously thought that BSF do not feed in the adult stage [14]. However, there is recent evidence to suggest that adult BSF benefit from feeding. Nakamura et al. reported that adult BSF longevity increased with sugar intake [42], whereas Bertinetti et al. [43] suggested that a protein-rich diet for adult BSF increases oviposition and longevity parameters. Bruno et al. [44], after investigating the adult BSF midgut, reported that the flies can ingest and digest food, with quality and quantity of food affecting longevity. These results collectively suggest that adult BSF do not rely exclusively on body nutrient and energy reserves accumulated during the larval stage.

BSFL are able to recycle various agricultural by-products [18, 45, 46]. However, the development time and various life-history traits of BSFL are highly dependent on the nutritional quality and nutrient balance of the feeding substrates. Consequently, BSFL performance parameters, such as prepupa wet weight, larval development time, survival rate, and protein and lipid content, differ among different substrates, such as concentrates and manure, food wastes, and sewage sludge (Table 1).

**Table 1:** A summary of literature values (ranges) for prepupae wet weight, duration of larval development, survival, and protein and fat content of black soldier fly larvae reared on different kinds of substrates

Items	Manure^1^	Feed concentrate^2^	By-products^3^	Sludge^4^
Rearing temperature, °C	9–32	20–35	20–35	21–28
Prepupae wet weight, mg	70–299	99–252	60–78	70–190
Larval development, d	30–34	15–24	19–30	15–20
Survival rate, %	74–93	81–93	80–98	39–76
Protein content, % of DM^5^	32–45	33–39	45–46	–
Lipid content, % of DM	18–33	31–35	20–31	–

^1^Based on references: [15, 47–51]

^2^Based on references: [45–47, 52, 53]

^3^Dried distillers grain, sugar beet pulp [18], vegetable by-products [18, 45, 46]

^4^Digested and undigested sludge [47], faecal sludge [54]

^5^In all of these studies nitrogen content was converted to crude protein content by multiplication with factor 6.25

Previous studies have demonstrated the successful use of BSFL to manage swine, chicken and cattle manure [14, 15, 47] and human faeces [48]. In the latter study, the effect of human faeces on BSFL was investigated with two different feeding regimes, different feeding ratios, and different larval densities. The authors observed that larvae fed a single lump amount of faeces had slower development and were larger than those fed every other day. The authors suggested that in response to the single lump amount, the larvae increased feed intake to compensate for nutrient deficiency [48]. The prolonged development time and increased larval weight support the hypothesis that reduced protein content in the diet causes increased larval feed intake. Also Lalander et al. observed that prepupal weight was not reduced, and the amino acid (AA) composition was even better in BSFL fed with human faeces, compared to poultry or dog feed [47]. They concluded that the nitrogen content of the substrate is of more importance than the content of easily available carbon compounds, e.g. found in fruit waste. Therefore, the diet should contain easily available carbon in combination with a sufficient content of protein [47].

Diener et al. reported that BSFL are capable of significantly reducing sludge biomass [49]. However, others showed that BSFL are not able to develop properly in pure faecal sludge and observed that BSFL developed much faster when food waste was added to the faecal sludge in a 50:50 ratio to enhance the nutritive value of the faecal sludge [12]. Lalander et al. [47] evaluated the development of BSFL on different kinds of urban organic waste and found that the larvae fed anaerobically digested sludge had the lowest growth rate, and the longest development time compared with undigested sludge. Based on these observations they suggested that the volatile solids content (sample compounds lost during combustion) may influence the size of the larvae, while the volatile solids and protein content together affect the development time of the larvae. In summary, the high substrate flexibility of BSFL and the resulting plasticity of the growth rate allow the successful development of BSF larvae on substrates of different nutrient qualities [48]. However, in this context it should be noted that European regulations of animal feeding, as laid down in the Commission Regulation (EU) 2017/893 [55], prohibit the use of e.g. catering waste, or manure and faeces as feed for insects.

## Growth potential, feed efficiency and greenhouse gas emission

As compared with other farmed animal species, rearing of insects is thought to be more sustainable because of their high growth rate and efficiency. In the following, we present a detailed comparison of data on growth performance, feed, energy and protein conversion efficiency in BSFL and other monogastric farmed animal species based on literature values (Table 2). Unlike conventional livestock such as chicken and swine, which are usually reared on standard diets adequate and balanced in nutrients, BSFL are fed a variety of diets with different nutrient compositions, ranging from chicken feed to organic waste products, which should be considered when deriving and comparing efficiency parameters. For a sound comparisons of BSFL with other livestock species, we first limited our comparisons to monogastric livestock species, as ruminants and BSFL can never compete for resources (e.g. for fibrous feed material). Second point was to select the most efficient farm animal species (i.e. chickens, pigs and fish) in order to understand the present upper limits of biological efficiency of different species in terms of converting plant biomass into animal biomass. Although it may be commercially relevant to compare BSFL with animals in less efficient production systems (e.g. those pigs or chickens that are not adequately fed or managed in certain countries or regions), such comparisons will imply a larger bias in favor of BSFL, when biological upper limits are to be explored.

**Table 2:** Mean fattening period, relative and specific growth rates, feed conversion ratio, crude protein and gross energy conversion ratio (fresh matter) of black soldier fly larvae vs. meat producing monogastric livestock^1^

Category	Fattening period, d	RGR^2^, %	SGR^3^, % per d	FCR^4^	PCR^5^, g CP/100 g BW gain	GECR^6^, MJ GE/100 g BW gain
Black soldier fly larvae	21	634,428	41.3	7.9	24.6	5.1
Chicken (broiler)	39	5802	10.5	1.7	30.9	2.6
Pig (pork)	158	7061	2.7	3.1	44.6	4.5
Fish (Atlantic salmon)	587	1,702,045	1.7	1.2	49.3	2.7

^1^*RGR*, relative growth rate; *SGR*, specific growth rate; *FCR*, feed conversion ratio; *PCR*, protein conversion ratio (g of crude protein needed to gain 100 g of body weight); *GECR,* gross energy conversion ratio (MJ of gross energy (*GE*) needed to gain 100 g of body weight). Equations to calculate RGR and SGR are based on [56, 57]. Values of initial and market body weight, body weight gain, feed intake, as well as protein and gross energy intake and further details of calculations and calculations for conversion of gross energy from species-specific metabolizable energy are presented in Additional file 1: Supplementary Materials 1. All raw data and calculations based on literature data are available in an Excel file stored in a repository (https://doi.org/10.5281/zenodo.5886206)

^2^RGR = ((market body weight (g) – initial body weight (g))/ initial body weight (g)) × 100

^3^SGR = ((ln (market body weight in g) – ln (initial body weight in g))/ fattening period (d)) × 100

^4^FCR = Feed intake (g) / body weight gain (g); feed intake is based on fresh matter

^5^PCR = Crude protein intake (g) / body weight gain (g) × 100; crude protein is N × 6.25

^6^GECR = Gross energy intake (MJ GE) / body weight gain (g) × 100

BSFL have a higher relative growth rate (RGR) than chicken and swine, but not fish (Table 2). Specific growth rate (SGR), a parameter describing relative growth within a given time period, is higher in BSFL than in terrestrial and aquatic livestock. The difference between SGR in BSFL and the nearest fast-growing species, i.e. broiler chickens, is about fourfold. The shorter larval fattening period (21 d) combined with the rapid growth potential (i.e. SGR) of BSFL suggest a high number of production cycles per year at farm level. In contrast, broiler production can have 4.1 to 7.4 cycles per year, depending on the production system [58], which means that the number of production cycles per year with BSFL is at least one order of magnitude higher.

**Fig. 2:**
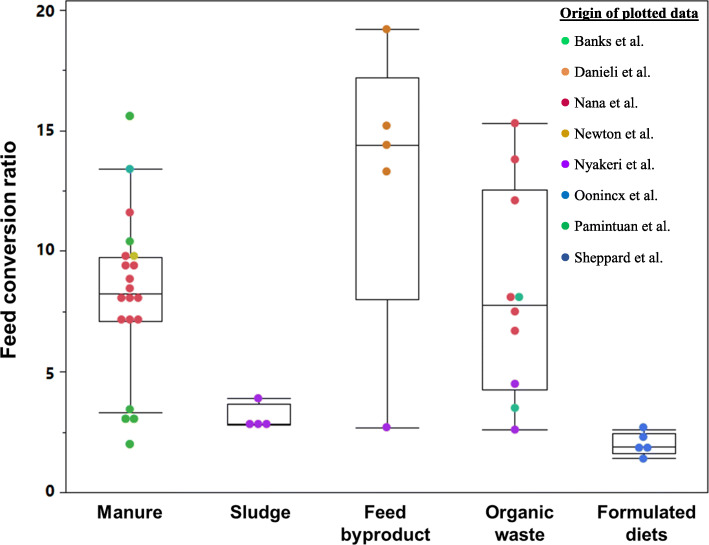
Substrate dependence of feed conversion ratio in black soldier fly larva reported in different studies. Formulated diets are nutrient adequate chicken and pig diets. Banks et al. [48], Danieli et al. [62], Nana et al. [63], Newton et al. [64], Nyakeri et al. [54], Oonincx et al. [65], Pamintuan et al. [66], Sheppard et al. [50]

There has been quite a discussion on how to assess feed efficiency for BSFL, which led to new indices in some cases [59, 60]. A common and simple definition of feed efficiency for fattening farmed animals is the body weight gain per unit of feed consumed [61] which is often expressed as feed conversion ratio (FCR). Salmon and chicken are the most efficient farmed animal species in terms of converting feed into body mass (Table 2). For BSFL, FCR can be highly variable as it depends on the feeding substrates (Fig. 2) with increasing FCR values if low protein diets are fed [48]. Considering the high moisture content (up to 70%) in feeding substrates of BSFL, the FCR does however not seem to be an appropriate conversion index to assess feed efficiency of BSFL in comparison to other species, whose diets contain much higher amounts of dry matter (DM, see literature values in  Additional file 1: Supplementary Materials 1). In order to make feed efficiency of BSFL better comparable to that of other farmed animal species, we calculated protein and gross energy conversion ratios (PCR and GECR) (Table 2). Protein and gross energy conversion ratios (PCR, GECR) were calculated as the amount of crude protein or gross energy consumed over the raising period to gain 100 g of body weight (BW) or 100 g of larval mass in the case of BSFL. The use of gross energy, i.e. total chemical energy in a given substrate, instead of the species-specific metabolizable energy in the calculations is particularly important as it allows a more objective comparison base for energy utilization efficiency. BSFL have the lowest PCR compared to the other farmed animal species (Table 2), implying the most efficient utilization of protein by BSFL. For conversion of dietary gross energy to body mass (i.e. GECR), chicken and fish are more efficient than BSFL. A main limitation of these comparisons is the estimations of nutrient and energy intakes. It is commonly thought that the amount of ingested feed can easily be determined for pigs and poultry. As reviewed by Patience et al. [61], it is the amount of disappeared feed what is being measured instead of the amount of feed consumed. However, the difference between the amount of feed consumed and the amount that disappears can be as high as 10% to 30% in pigs [61], implying the measurement of feed intake may be inaccurate even in one of the best studied farm animals. Determining the feed intake of BSFL living in their substrate is a much greater challenge. Furthermore, the present calculations do not consider body composition of different animal species, which differs substantially in moisture, nutrient and gross energy contents.

For insects the estimation of the efficiency of conversion of the ingested food (ECI) based on dry matter is relatively widespread (ECI = B/(W − R) × 100%, where B = total larval + pupal biomass at harvest minus total larvae biomass at start (g); W = total amount of substrate provided; R = residual of the substrate) [59, 60]. Although the ECI takes into account the amount of feed that disappears due to the consumption of larvae, i.e. amount of ingested feed, the rearing substrate residue consists not only of uneaten substrate but also includes insect frass, cuticle moults and microbial and fungal material. Therefore, ECI is dependent on the composition and the microbial colonization of the substrate, and thus also has limitations.

It should be considered that the conceived advantage of BSFL having an environmental footprint better than conventional livestock [67, 68] is only true if BSFL is produced on organic waste and its protein would directly be used for human consumption. This is a key issue and implies that BSFL should not be considered a novel livestock species in competition with conventional ones, but as a complementary species that can efficiently utilize low-grade feedstuff and organic waste that cannot be utilized by other livestock, and thus contribute to closed nutrient cycles in agro-ecological systems. However, due to current regulatory requirements [55], insect protein can only be produced on substrates approved for other livestock such as chicken and pigs. Thus, under current legislation, if BSFL components are used in animal feed its environmental footprint ought to be compared with that of plant-derived feedstuffs or a double transformation loss needs to be considered.

Information on gas exchange and emissions is important to understand energy metabolism and energy efficiency of BSFL and to design optimal diets to achieve high efficiency in insect farming. Sustainability and environmental impact of insect production and waste management via vermicomposting through BSFL is considered favorable compared to protein production with conventional livestock and the conventional composting of food waste [69, 70]. The reduction of gaseous emissions by using BSFL in biowaste management adds to the sustainability of BSFL production [71]. Nevertheless, Chen et al. [72] demonstrated that, compared to conventional composting, methane, dinitrogen monoxide and ammonia emissions were reduced but CO_2_ production was increased by converting pig manure with BSFL. However, direct greenhouse gas (GHG) and nitrogenous gas emission data derived from insect production are currently limited [67, 73, 74].

Short-term experiments lasting for few hours were conducted to determine CO_2_ production, oxygen (O_2_) consumption or respiratory patterns in adult insects [75–77] (Additional file 2: Supplementary Table 1). However, data on the analysis of continuous gas exchange in BSFL is scarce. As to be expected fed larvae show a much higher hourly CO_2_ production ranging from 2.3 to 63.1 μL/mg larval mass than those measured without feed (0.3 to 4.9 μL/mg larval mass) (Additional file 2: Supplementary Table 1). Recently, Sandrock et al. [74] provided data on CO_2_ and methane production in BSFL. Different to the other studies on continuous gas exchange measurement in insects (e.g., [78, 79]) they showed gas production not for individual animals but the combination of BSFL with their substrates over a growth period of 14 d. The authors found that the magnitude of gas production was strictly dependent on feeding events with a CO_2_ production peak occurring immediately after feed supply. Parodi et al. provided data comparing gas production from pig manure vs. pig manure incubated with BSFL and found increased CO_2_ and ammonia but not methane production, when larvae were present [71]. The authors concluded that Archaea resident in the manure are responsible for methane production but not BSFL. The increasing pH over time shifted the NH_4_^+^/NH_3_ equilibrium in favor of NH_3_. However, at increasing pH the CO_2_ production decreased as a sign of improved feed efficiency when larvae are present. Methane production as a sink for produced hydrogen increased at higher pH as indicator for microbial activity but also as a sign of better accessibility of nutrients for the larvae. The reduction of CO_2_ production at higher pH could be therefore at least partly linked with the production of methane.

We analyzed CO_2_ production and O_2_ consumption of BSFL within their substrate (chicken feed) and under starvation conditions using continuous gas exchange measurement in respiration chambers (see Table 3 for further details). The gas exchange differed largely due to the metabolic state with larvae O_2_ consumption and CO_2_ production being 4 and 3 times lower when starved (Table 3), respectively. Because CO_2_ is also produced when incubating feeding substrate without BSFL, the gas exchange measured in BSFL also results from microbial metabolism in the feeding substrate in addition to the nutrient metabolism of the larvae [80]. It is however difficult to quantify the microbial contribution to the gas exchange of BSFL in their substrate because the presence of the larvae in the substrate modifies the substrate microbiota in itself, and thus their contribution to the total gas exchange.

**Table 3:** CO_2_ production and O_2_ consumption of black soldier fly larvae (5^th^–6^th^ instar) fed on chicken feed for three subsequent days or during starvation for one day^1^

Gas exchange	Ambient temperature, °C	Changes in larval BW in relation to starting BW, %	Gas exchange on measurement d^b^, μL/mg BW/h			
Experiment (Fed)^c^	27.5	+ 119.6	1	2	3	SE
CO_2_ production			2.21	1.88	2.33	0.17
O_2_ consumption			1.71	1.71	2.17	0.13
RQ^d^			1.29	1.09	1.07	0.10
Experiment (Starved)^e^	32	− 15.4				
CO_2_ production			0.53	–	–	0.03
O_2_ consumption			0.71	–	–	0.03
RQ			0.75	–	–	0.01

^a^All larvae were measured in vessels with 23 cm^2^ surface area, a volume of 536 mL placed inside respiration chambers. The ventilation rate was 37.2 L fresh air/h per respiration chamber. The respiration chambers were placed inside a climate controlled closet (3 chambers per closet) kept at the indicated temperatures in the dark. Continuous measurement of gas exchange was performed at 21 min intervals using open-circuit indirect calorimetry. CO_2_ and O_2_ concentrations were measured by infrared absorption and paramagnetic gas analyzers (Maihak AG, Hamburg, Germany), respectively. CO_2_ and O_2_ concentrations were multiplied with air flow in and out of the chambers and normalized to BW and time to obtain CO_2_ production and O_2_ consumption. We thank Hermetia Baruth GmbH for their cooperation in setting up a black soldier fly colony

^b^Gas exchange data was related to the mean BW at d 3 (fed) of the respiration measurement period or the mean of start and end BW of the 1 d respiration measurement period (starved)

^c^Fed status (*n* = 6) = 150 larvae at 14.5 d after hatching with a mean BW of 108.4 mg at start of the measurements were fed on 114 g chicken feed substrate. The vessels were filled one d before the start of the experiment with 34.2 g chicken starter feed and 79.8 g of water (30% feed:70% water (w/v)). After 3 d the gas exchange measurement was stopped and the larvae were isolated from the frass, counted, cleaned with tap water, and dried with paper towels. Afterwards, their wet BW was determined

^d^RQ = Respiratory quotient: CO_2_ production (μL)/O_2_ production (μL)

^e^Starved status (*n* = 6) = 150 larvae at 18 d after hatching. The larvae were grown until 18 d, transferred to the insect vessels on d 18 at a mean BW of 193.3 mg, and measured in the respiratory chambers for one d without feed. The wet BW was determined as above

The respiratory quotient (RQ) is defined as the ratio of CO_2_ production over O_2_ consumption rates and provides information on the type of nutrient oxidized or stored. Thus, an RQ of 1 and 0.7 reflects pure glucose oxidation and pure fat oxidation, respectively [81]. Consequently, we observed an RQ value close to 0.7 in starved BSFL oxidizing their body fat reserves (Table 3). An RQ value larger than 1 in fed BSFL at post-hatching d 15 indicates fat synthesis [82], which decreased as they approached the pupal stage (Table 3). This observation is consistent with the observation of increasing body fat content from 4.8% at hatching to 28.4% 14 d post-hatching [83].

The values of CO_2_ production during resting (0.26–4.9 μL/mg insect mass per h) of insects (i.e. for adult flies, the experimental setup prevented flying and feeding) derived from literature (Additional file 2: Supplementary Table 1) [84] were largely comparable to conventional livestock (lactating dairy cow: 0.34 to 0.42 [85]; lactating dairy cows at 5 and 42 weeks of lactation: 0.33 and 0.34 μL/mg BW per hour [86]; horse: 0.17 μL/mg BW per hour [87]). Pang et al. demonstrated that increasing the pH of the initial substrate effectively accelerated the BSFL growth and decreased CO_2_ emissions, while simultaneously increasing NH_3_ emission [67]. The increase of pH is equivalent to the removal of protons and thus the NH_4_^+^/NH_3_-equilibrium in the substrate shifts towards NH_3_ production. This increases the nitrogen incorporation resulting in higher body protein accretion which in parallel increases carbon fixation in the body and reduces CO_2_ production.

In the future, more data are needed on continuous gas exchange in BSFL to calculate energy balance, heat production, and direct greenhouse gas emissions from BSFL and to better understand how efficiently nutrients from different substrates are utilized.

## Intestinal biology

Over the last decades, our increasing understanding of the digestive tract physiology of farmed animals has led to significant gains in productive performance. However, despite the growing interest in BSFL bioconversion of organic waste [17, 20, 88], our knowledge about the morpho-functional and biological features of the gastrointestinal tract (GIT) of BSFL is still limited. The functionality of the GIT is a major factor affecting the growth rate of an animal. The shared microbial habitat created by BSFL, their GIT microbiota and the microbiota of their substrate, might improve metabolic efficiency of BSFL by prioritizing carbohydrate or sulphur compound metabolism inside the substrate [80]. The microbial cooperation in digestive processes [80], together with the morpho-functional organization, especially of the midgut, could play a role in the efficiency of feed energy conversion in BSFL. Eventually, all of these aspects may result in the higher growth rate of this insect species compared to other farmed animal species.

### Intestinal morphology and digestive functions

**Fig. 3:**
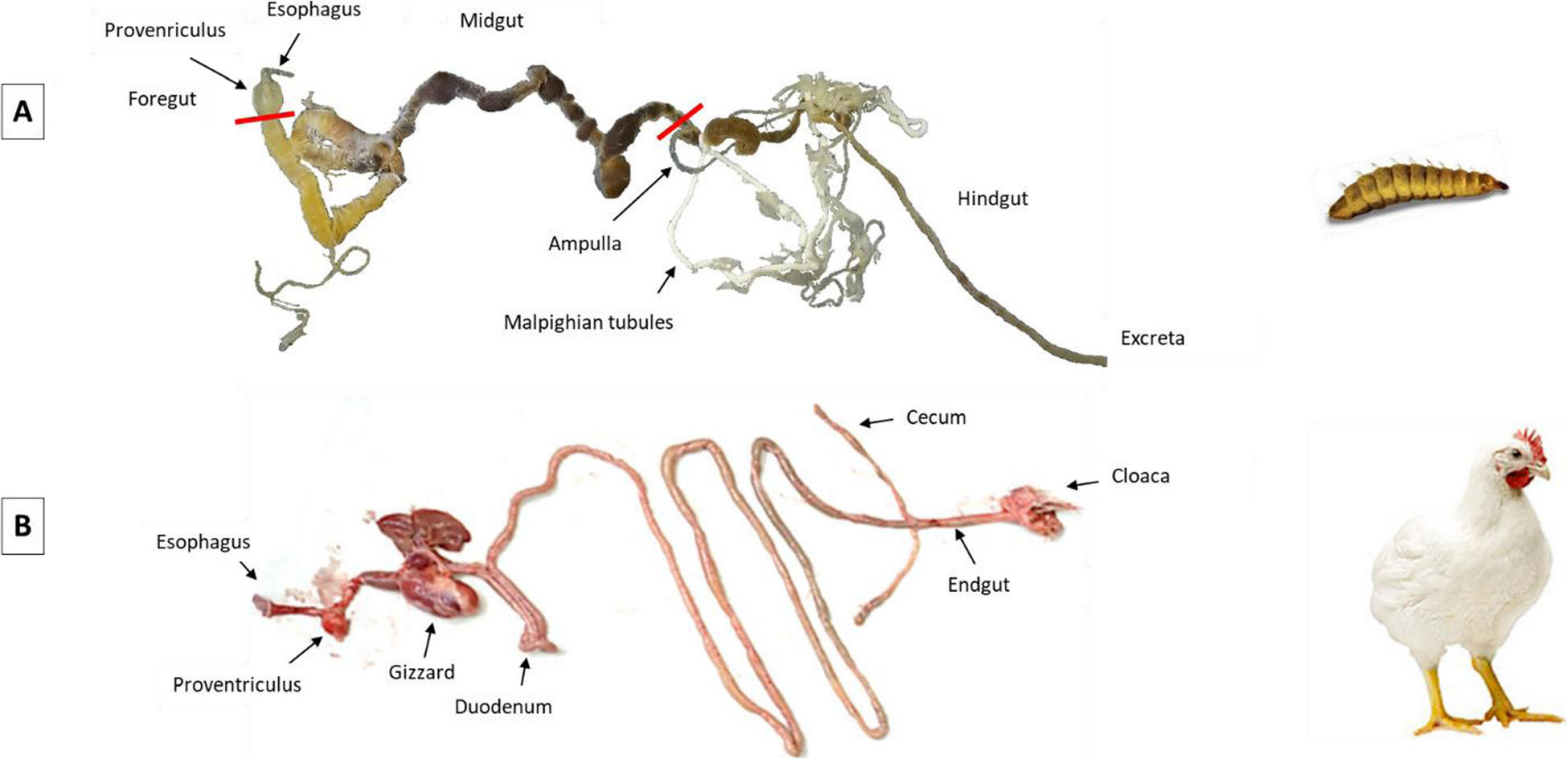
Digestive tracts of a black soldier fly larva (**A**) and a chicken (**B**). In BSFL, feed passes through the esophagus to the proventriculus and into the midgut (red line: discrimination of the midgut). The ampulla is the entry of the Malpighian tubules in the gut and separates midgut and hindgut

Compared to conventional livestock species, data on the physiology of the intestine of BSFL are scarce. Considering that chicken is the most efficient terrestrial farmed animal species, but needs high quality feed that can be directly consumed by humans, we compare chicken GIT with that of insects (Fig. 3). As compared to mammals, such as dairy cows or pigs [89] the GIT of chickens [90], is much shorter relative to the body length. It consists of the esophagus, crop, proventriculus, gizzard, duodenum, jejunum, ileum, cecum, colon, and cloaca (Fig. 3). In poultry, feed ingredients are largely digested enzymatically, and because of the short GIT they require feed which has a greater energy and nutrient density compared to pigs and ruminants. Although microorganisms in poultry colonize the entire length of the GIT, the highest microbial densities and greatest species diversity are found in post gastric regions, particularly in the distal gut of poultry (i.e., ceaca and colon). In the distal gut of poultry, the microbiota produces short-chain fatty acids (SCFA) mainly from non-starch-polysaccharides indigestible by the host, which can be utilized as energy source [91]. Caecal fermentation provides approximately 3% to 11% of the energy as SCFA in chicken [92].

The digestive tract of insects starts with the mouth apparatus. Kim et al. [93] reported a head capsule width of between 0.1 and 1.1 mm in 1^st^ to 6^th^ instar BSFL which is of similar magnitude as the preferred particle size of about 0.15 mm suggested by Liland et al. [41]. The BSFL mouth part has a well-developed mandibular-maxillary complex that contains a sweeping apparatus similar to those of scavengers’ larvae to improve the efficiency of uptake of organic materials. The maxillary rasp as well as the lacinial teeth of the larval mouth apparatus are used to grind the particles picked up from the semiliquid substrate. Subsequently, pretreated particles are transported to the pharynx after sorting, reaching the “grinding mill” where they are further crushed [36]. Following the mouth part, the intestine is divided into three main parts, which can be further subdivided into different functional units [93]. The three-compartment organization of the digestive system is conserved in all insects [94]. As also found in BSFL, the GIT consists of i) the foregut, the anterior part of the alimentary canal involved in food ingestion, storage and disintegration, ii) the midgut, where food digestion and nutrient absorption take place, and iii) the hindgut (Fig. 3). The last part of the GIT of BSFL is responsible for the reabsorption of water and ions coming from the hindgut contents and urine derived from Malpighian tubules which enter the hindgut [95–97]. However, as discussed [98] also AA are absorbed by the hindgut, coming from Malpighian tubules. Amino acid uptake in the hindgut of insects differs from the generally accepted concept in vertebrates, in which no amino acid uptake takes place in this intestinal compartment.

In contrast to foregut and hindgut fermenting mammals and wood-feeding hindgut fermenting insects like termites [96], BSFL appear to be midgut fermenters. In this regard, Bonelli et al. [97] and Bruno et al. [99] presented data on midgut morpho-functional regionalization shaping the digestive enzyme activity and the residing microbiota. Bruno et al. showed that the anterior part of the midgut had the highest microbial diversity that gradually decreased along the midgut [99]. However, bacterial communities can be found in all parts of the BSFL gut, as reviewed by De Smet et al. [100]. To our knowledge, whether microbes in the GIT of BSFL produce SCFA has not yet been investigated, but for termites and honey bees SCFA production in the hindgut and metabolization has been demonstrated [101, 102]. However, it has been shown that BSFL can remove and metabolize SCFA such as propionic, butanoic and pentanoic acid from their substrate [103].

The individual regions of the midgut have specific luminal pH values with particular characteristics at both the structural and functional levels, as well as differences in the microbial density and composition of the microbiota [99]. The anterior region of the midgut showed a pH value of around 6 and an incipient acidification of the intestinal contents takes place resulting in a pH of about 2 in the second part of the midgut region. In the posterior region of this gut segment, the pH is alkaline (mean pH 8.3) [97].

In the common fruit fly (*Drosophila melanogaster)* the so-called copper cells accumulate copper and are involved in acidification to pH 2 of the lumen in the anterior part of the midgut, as has also been reported for BSFL [104]. There are a number of similarities between copper cells and the acid-producing gastric parietal cells of the mammalian stomach [104]. In contrast to the BSFL [97], Bruno et al. could not identify the presence of copper cells in the intestinal epithelium of adult BSF [44]. This could be the reason why no peritrophic matrix was found in adult BSF, in contrast to the BSFL, a mechanical protection of the intestinal epithelium [44]. This selective permeable membrane surrounds the ingested feeds in the midgut of BSFL, preventing a direct physical contact with the entodermal microvillar cells [96]. An important role of the peritrophic matrix in increasing digestive efficiency was shown in larvae of the Indian meal moth *Plodia interpunctella* [105]. We assume that this is a general mechanism also present in the BSFL. The midgut of BSF consists of a monolayered epithelium, mainly formed by columnar cells in the adults, which show a different thickness in the various regions of the intestine, characterized by a basal infolding and apical microvilli. Glycogen granules and the rough endoplasmic reticulum are visible in the cytoplasm of the epithelial cells [44].

Crucial physiological adaptations occur in the gut of BSFL as response to dietary components. For instance, the length of microvilli in the posterior midgut of BSFL increases in response to a diet with low nutrient density [106], implying an adaptation to increase absorption efficiency in the gut. Furthermore, Gold et al. [107] found that diet residence time in the different midgut regions of BSFL is influenced by the protein and non-fibre carbohydrate content in the diet.

### Digestive enzymes

The ability of BSFL to digest different kinds of organic matter is related to the enzymes of the salivary gland and especially the GIT [108]. The main digestive enzymes of the digestive tract found in BSFL are amylases, lipases and trypsin- and chymotrypsin-like proteases which support the observation that it belongs to the polyphagous insect group [108–110]. Compared to the larvae of the house fly (*Musca domestica*)  BSFL showed higher amounts and activities for leucine arylamidase, β-galactosidase and α-fucosidase in saliva as well as α-galactosidase, α-mannosidase and α-fucosidase in the gut [108]. Specific enzyme activities are pH dependent. Lee et al. and Song et al. found an α-galactosidase and an endo-1,4-β-mannanase, respectively, in intestinal microorganisms from *H. illucens* [111, 112]. They expressed the recombinant proteins, rAgas2 and rManEM17 in *E. coli*, and found highest activity at 40 °C and pH 7.0 (rAgas2) and at 55 °C and pH 6.5 (rManEM17), respectively. In the anterior region of the midgut the highest activity of soluble amylase but also some lipase activity was observed [97]. In the middle part of the midgut lysozyme activity was found, besides the main activity of lipase as well as trypsin-like and chymotrypsin-like endopeptidases. In the posterior region of the midgut, mostly lipase activity was detected [97]. Polyphagous insects are able to synthesize a wide range of proteolytic enzymes for digesting the diverse proteins obtained from several kinds of foods. For example, a chymotrypsin- and a trypsin-like protease has been characterized in BSFL showing spatially and temporally different expression pattern [109].

Digestive enzymes activities vary depending on the diet [113], gut environment, pH [114], and ambient temperature [97]. Anti-nutritive factors in the substrate may also play a role in digestive efficiency as shown for enzyme inhibitors derived from wheat and barley that affect α-amylase and trypsine-like activities in *Spodoptera frugiperda* [115]. For BSFL fed cottonseed press cake, Tegtmeier et al. [116] reported the adaptation of their intestinal microbiota to metabolize gossypol, a sesquiterpene known to inhibit lactate dehydrogenase. However, more specific data on anti-nutritive factors affecting digestive enzymes in BSFL are lacking. During the feeding process of the larvae, the substrate temperature can reach as much as 45 °C, which is the optimum temperature for proteolytic activity at a pH of ~ 8 in the posterior midgut of BSFL, and strongly contributes to the efficiency of BSFL in substrate bioconversion [97, 108]. Also Meneguz et al. [117] suggested a link between basic pH and protease activity. They showed that the BSFL can alkalinize their substrate to a pH of 9.4 and suggested that the pH of the environment may improve the growth of specific bacteria, which could benefit larval development. To the best of our knowledge information on specific transport systems for nutrient uptake and absorption is not available yet for BSF larvae and adults.

### Intestinal microbes and fiber digestion

Conventional vertebrate livestock but also insects such as honey bee or German cockroach harbor a wide range of different microorganisms, including bacteria, protozoa, fungi, archaea, and viruses in their GIT. In insects these may have different effects on the host, including the provision of necessary nutrients such as vitamins, the stimulation of the immune system, removal of pathogenic microorganisms, sex determination, hormonal signaling, and behavior [102, 118]. In nature, there are different processes for decomposition of lignocellulose that rely on microbial communities producing a variety of lignocellulolytic enzymes. The intestinal microbiota of different insect species using foliage, wood or detritus as substrate are important for the degradation of organic material like lignin, and cellulose [119–121].

The GIT of BSFL harbors various groups of microorganisms, which play several functional roles, and enables the larvae to digest and use specific dietary compounds efficiently [80] for the accretion of body mass. The first study characterizing the gut microbial community of BSFL, reared on three different substrates (food waste, calf forage, and cooked rice), was published by Jeon et al. [122]. In this metagenomics study, the bacterial communities in the gut of BSFL were analyzed by pyrosequencing of larval intestinal DNA. The authors reported that the features of the unique bacterial community in the BSFL gut can be modified by changing the diet [122]. This was also demonstrated for the different segments of the midgut, using different substrates, e.g. fish vs. mixed vegetables [99]. Also the level of the mycobiota in the diet as well as the exposure time affect the biodiversity. Therefore, a transient and environment-dependent composition of the mycobiota was suggested [123]. Although Wynants et al. indicated that the microbial quality and community composition of BSFL is associated with the microbial composition of the substrate [88], they concluded from their results the possible presence of a microbial core community albeit with variable abundance. In contrast, Klammsteiner et al. [124] observed little influence of the diet type on BSFL gut microbiome and proposed a core microbiota of low abundance taxa (e.g. *Actinomyces* spp.*, Dysgonomonas* spp.*, and Enterococcus* spp.) providing functions responsible for growing of BSFL in various environments. Thus, it is likely that the GIT of BSFL contains a core microbiota, but further studies are needed to identify the species involved and their proportion.

The digestion of dairy manure by BSFL resulted in reduced cellulose and hemicellulose levels [125]. The recently sequenced microbiota of BSFL shows that lignocellulolytic potential is present [126]. Ur Rehman et al. [127] demonstrated that BSFL digesting a mixture of chicken and dairy cow manure have a higher growth and cellulose degradation compared to cow manure only. These authors also revealed changes in the fiber structure, resulting in a lower carbon/nitrogen (C/N) ratio. Cellulolytic activity in BSFL was also demonstrated by Supriyatna and Ukit [128]. They isolated intestinal bacteria of BSFL and found by biochemical differentiation that *Bacillus* spp. had the highest cellulolytic potential of the isolates with highest activity at pH 7 to 8 and 40 °C. Using a metagenomics approach *Dysgonomonas* sp. were identified by Klammsteiner et al. [129] and Jiang et al. [80]. The same methodology was used to identify a cellulase (CS10) in BSFL that belongs to the glycosyl-hydrolase family 5 with one catalytic domain, broad pH spectrum and optimal activity at pH 6 and 50 °C [130]. Recently, Intayung et al. reported cellulose activity in BSFL decreasing from 6 to 18 days of age [110]. Also rice straw, rich in cellulose and hemicelluloses but also lignin can be converted by BSFL when mixed with solid restaurant waste to improve the nutrient balance [131]. Therefore, it can be assumed that BSFL are capable of degrading fibers, but the extent to which this is possible requires further investigation. In addition, the supplementation with a mix of specific bacteria in addition to various enzymes further increased lipid synthesis by BSFL [131]. Mazza et al. reported that BSFL reared in chicken manure together with *Bacillus subtilis* derived from the BSFL gut and a bacteria mix harvested from BSF eggs improved larval mass gain [132]. Comparable data exist also for co-conversion using yeast. It was shown that *Saccharomyces cerevisiae* improves the conversion of coconut endosperm waste by BSFL, thereby increasing the lipid output [133]. Thus, the addition of microorganisms or enzymes to the feed substrate may have a positive effect on growth and feed conversion of BSFL. 

## Nutrient utilization and requirements

Animals eat to obtain energy and nutrients to meet demands for maintenance, growth, and reproduction. Nutrients of particular importance are those which cannot be synthesized in the animal’s body or cannot be synthesized in sufficient amounts during critical physiological state e.g. in very young animals or ontogenetic periods of high demand for certain nutrients. Thus, certain amino and fatty acids as well as vitamins and minerals are indispensable. In this regard, insects do not differ from other animal species as they must acquire indispensable nutrients from the environment via digestion [94]. However, in contrast to conventional farmed animals for which research on their nutritional needs goes back more than a century (e.g. [134]), knowledge on nutrient requirements for insects is rather limited. Over the decades breeding and genetic selection have improved efficiency and growth rates of conventional livestock with the consequence that highly specialized and high performing animals have been created that require more nutrient and energy dense diets to meet their demands. For example, the high growth rate and laying performance of modern broiler and layer strains can only be ensured with properly formulated rations that are protein-, energy- and mineral-dense and are tailored to meet the age-specific nutrient demands to avoid large nutrient losses and environmental pollution [135]. It should be noted here that although the larval life span is shorter when compared to conventional farmed animals, the nutrient requirement likely differs along ontogenetic sequence. For example, lipid reserves are frequently deposited in later instars in insects (summarized by Scriber and Slansky [136]). However, this is different in BSFL larvae in which lipid deposition starts shortly after hatching [83] as discussed in detail below.

For saprophagic insects like BSFL that just only recently came into focus as a mini-livestock, research results on nutrient demands are still scarce. Likewise, relevant information regarding the nutrient requirements of other Diptera such as *D. melanogaster* are still missing [137]. Larvae of BSF can feed on a large variety of substrates, from high quality feeds such as cereals to vegetable, fruit and slaughter wastes and even manure. This indicates their flexibility to utilize a wide range of proteins and carbohydrates edible by humans but even can tolerate dietary ingredients which are indigestible or even harmful to other species [26, 107]. However, as for conventional farmed animals, in insects a nutrient imbalance also results in higher catabolism and excretion which can reduce growth. Scriber and Slansky [136] reviewed studies showing that insect larvae can deal with the reduced content of a nutrient by increasing feed intake and simultaneous change of efficiency. Interestingly, BSFL might also depend on the associated microbiota in their substrate to obtain certain nutrients such as vitamins or sterols, because sterile BSFL did not grow well on autoclaved diets [138]. Insects follow three strategies to handle the disadvantages of imbalanced diets: changing the total amount of ingested substrate; migrating to a substrate with a different composition, or regulating the efficiency of nutrient conversion [139]. In contrast, when feed with properly balanced nutrients is available fast growth is a common strategy of insects to pass through e.g., stages of high morbidity or food shortage [136]. The reader is referred to the studies of Barragan-Fonseca [140] for strategies of BSFL to deal with different nutrient concentrations and dietary imbalance.

In classical animal nutrition energy and nutrient requirements are estimated by empirical and factorial methods, (e.g., [141]). In general, energy and nutrient requirements are estimated for maximum performance in response to varying energy and nutrient intakes or the amount of energy or nutrients required for a certain function or parameter (e.g. growth, milk production, nitrogen balance). Usually, methods to measure nutrient requirements in conventional farmed animals consider one nutrient at a time in iso-nitrogenous and iso-energetic diets with graded levels of the nutrient in question being fed to determine the concentration where nutrient demand is met (e.g. [142]). Unlike conventional farmed animals, BSFL live in their feeding substrate, making it difficult to determine digestibility of feed, an important prerequisite for determining nutrient requirements and utilization efficiency. In fact, it is difficult to accurately determine the amount of feed actually ingested by the larvae, and coprophagy would result in apparently higher digestibility, due to microbes living in the substrate [136]. Gold et al. [138] were the first to develop a device to collect frass from individual BSFL and analyze its composition to measure digestibility of nutrients on the faecal level. For conventional farmed animals digestibility and nutrient requirements measurements on the faecal level have been abandoned because nutrients are absorbed from the small intestine, and digesta conversion in the large intestine are mostly of low net nutritive value for the animals [143]. Taken together, the methods used in conventional livestock to determine the nutritional requirements have very limited applicability to BSFL. In this context, measures of feed conversion ratios and efficiencies in BSFL should be viewed with caution due to the limitations mentioned above regarding proper measurements of feed intake and excrements production.

It has been long known that nutrients and energy in the diet need to be delivered in the right ratio to each other [144]. Following this principle, Raubenheimer et al. [145] developed geometrical framework methods for evaluating how different nutrients do interact in supporting a specific trait such as growth or egg yield. This method allows to determine the responses of traits to the relative proportions of components (nutrients) at a given stage of development. Interestingly, this method has been used quite frequently in insects [146–148], but only rarely in conventional livestock [149].

Recognizing that data on BSFL nutrient requirements are largely lacking, the following section reviews the available literature with respect to maximum and minimum levels of macronutrients (protein (or N), fat, carbohydrates) and minerals to support maximal larvae weight gain or optimal life-history traits of BSFL.

### Protein and amino acids

In insects, the dietary nitrogen and water content seem to predict the upper limits of larval performance [136], which might be related to the poikilothermic nature of this animal class. It was demonstrated that BSFL but also Argentinian cockroach utilize protein more efficiently than yellow mealworm and house crickets [59]. Different to the other analyzed insect species, the FCR in BSFL tended to be higher using lower protein diets (12.9% and 14.4% CP) than high protein diets (21.9% and 22.9% CP) [59]. The dietary protein or the nitrogen content of feed was identified as the most important factor affecting BSFL performance when fed diets including sorghum or cowpeas as well as manure from different animal species as substrate [65, 150].

Most insects besides e.g. cockroaches [151] have a need for the same set of 9 or 10 essential amino acids [139, 152] as in other farmed animals such as swine [141]. For example, as summarized by Genc et al. [139] tyrosine is necessary for the sclerotization of the cuticle and tryptophan for the synthesis of visual and screening pigments. It was recently shown, that supplementation of lysine to 3% over a basal concentration of 0.3% in the feeding substrate did not improve body mass gain but reduced larvae survival and development [153]. In general, insects contain relatively high amounts of lysine, threonine and methionine, which are major limiting essential amino acids in cereal- and legume-based diets for pigs and poultry [154]. However, quantitative requirement of essential amino acids is largely unknown for BSFL.

It is well recognized that in chickens, the growth rate and feed utilization efficiency depend on dietary protein level [155]. Similarly, protein content in BSFL diet is a key parameter that drives larval development and survival. In general, it appears that BSFL on substrates with higher protein content (22% of DM) than low protein content (13% of DM) achieve a higher larval weight, better bioconversion and feed conversion ratio, improved larval protein and lipid content, and reduced developmental time [45, 59, 150]. However, when reared on a diet low in protein (14% of DM) and lipid (2% of DM) but higher in carbohydrates (fruit vs. vegetable), the lipid content of BSFL was 1.4 times higher than when reared on a diet higher in protein content, but BSFL development takes longer (37 d instead of 21 d) [59]. Although dietary protein concentration is a key parameter for larval development and survival, there appears to be an upper threshold beyond which high dietary protein could be detrimental. When the dietary protein content exceeded 37%, toxic effects were evident and survival rate and adult emergence were impaired [140]. According to Barragan-Fonseca et al. it seems that the protein content of the larvae is regulated within narrow limits [46].

In addition to the quantity of proteins, the quality of protein (AA composition and digestibility) is also of critical importance [26, 156]. Previous studies reported that BSFL grown on a nutrient dense and amino acid balanced diet such as chicken feed show a shorter development time than when grown on a bread or a cookie and bread diet [59]. It was suggested that diets that have a similar AA composition to that of the BSFL, such as chicken feed compared to vegetable waste, resulted in a shorter developmental time of BSFL [113]. Thus, it is likely that mixtures of different organic waste and by-products with different AA patterns that complement each other could increase performance of BSFL. In this context, Gold et al. [157] reported that biowaste mixtures with similar protein and non-fiber carbohydrate contents of approximately 1:1, with protein and non-fiber carbohydrate ranges maintained between 14% to 19% and 13% to 15% (DM basis), respectively, resulted in better performance and less variability compared to individual biowaste sources.

Similar to conventional livestock also for BSFL the digestibility of dietary proteins is a determinant of protein quality [113]. However, knowledge on dietary protein digestibility as well as on presence and function of specific AA transporters as described for *Drosophila melanogaster* [158] is lacking for BSFL. Thus, there is still a major knowledge gap when it comes to the definition of AA requirements of BSFL. Recent data indicate that C/N ratio of BSFL feeding substrate is of crucial importance in terms of larval growth [46]. Beesigamukama et al. found a rearing substrate with C/N ratio of 15 being the most suitable for BSF larval yield [159]. This ratio is further supported by the results of Palma et al., who found that decreasing C/N from 49 to 16 resulted in an increased larval growth and yield by 31% and 51%, respectively [160].

### Lipids and fatty acids

The lipid content of BSFL is relatively high and differs during different stages of larval development [83, 161]. Depending on the diet, the lipid content ranges between 11% and 58% of DM [154] and is dominated by saturated fatty acids with up to 76% of total fat [162]. According to Makkar et al. [154] BSFL and prepupae contain 58% to 72% saturated fatty acids and 19% to 40% mono- and poly-unsaturated fatty acids which is however highly variable and dependent on the fatty acid composition of the substrates. Among the saturated fatty acids BSFL contain relatively high proportions (40% and more) of the medium-chain fatty acids lauric acid (C12:0) and myristic acid (C14:0) [163].

Along with glycogen, lipids represent essential energy reservoirs, and insects partly derive it from de novo lipogenesis, which mainly occurs in the fat body, and dietary lipid digestion and absorption in the midgut [106]. In a D_2_O labelling experiment with BSFL, decanoic (C10:0), C12:0 and C14:0 acid were present exclusively in their deuterated form, whereas palmitic (C16:0), palmitoleic (C16:1) and oleic (C18:1n9) acid were either deuterated or undeuterated, indicating that BSFL can produce these fatty acids in part via biosynthesis pathways and not only by bioaccumulation from the diet [163]. In BSFL, a large proportion of dietary fatty acids are converted to C12:0, which is the most prominent fatty acid in BSFL [154] with an amount of up to 52% of total fat [162]. Its relatively high melting point (43 °C) allows survival at ambient temperatures of 40 °C and higher, as occurs during substrate fermentation [164].

Substrate lipids can already be degraded in the substrate or they are digested and absorbed in the larval gut to free fatty acids and mono- and di-glycerides. High amounts of lipids and lack of carbohydrates in substrates such as fish offal (6.6% lipids) compared to manure (0.15% lipid) was found to increase larval developmental time until adult emergence and increase mortality [45]. The composition of dietary lipids directly influences the fatty acid composition of BSFL [41, 165]. Feeding fish offal as part of the diet increased n-3 fatty acid content up to 3% of total lipids. Using extracted flax cake (60% of the diet) or flaxseed oil (2% or 4% of the diet) in the feeding substrate of BSFL, resulted in 6% and 9.7% linolenic acid (C18:3n-3) in total fat, respectively [163, 166]. There seems to be a limit to the bioaccumulation of poly-unsaturated fatty acids (PUFA) by BSFL because approximately two thirds of PUFA were converted to saturated fatty acids [163].

Several authors [41, 162, 163] reported that BSFL are not able to synthesize PUFA which indicates that PUFA might be indispensable for BSFL similar to conventional livestock. Ewald et al. [162] indicated that relatively high levels of n-3 fatty acids in BSFL such as eicosapentaenoic acid (C20:5n-3) or docosahexaenoic acid (C22:6n-3) are due to their content in the rearing substrate. With progressing larval development the C12:0 synthesis is increasing, while the relative content of n-3 fatty acids decreases. Recently, the BSFL lipid metabolism associated transcriptome was deciphered [161]. The expression profiles of metabolic enzymes that are involved in the biosynthesis of acetyl-CoA, fatty acids and triacylglycerol were analyzed to investigate the regulation of lipid accumulation during BSFL development [161]. The result of this study showed that many genes that are involved in the rapid accumulation of short-chain fatty acids are highly expressed during early (1–4 d) and late developmental stages, whereas triacylglyceride deposition occurs mainly in the late stages [161]. Taken together linoleic acid (C18:2n-6) and C18:3n-3 might be indispensable to BSFL and need to be delivered by the substrate albeit the requirement level is not known.

### Carbohydrates

In livestock diets, carbohydrates provide well over one-half of the energy needed for maintenance, growth, and reproduction. Feed carbohydrates are composed of sugar monomers, and glucose is the most important source of energy for many animal tissues [167]. Also in insect species like BSFL, carbohydrates are a major source of energy [168]. However, they are not essential because, they can be synthesized from lipids (glycerol) or certain AA. Insects, such as the fruit fly can synthesize sugars de novo by gluconeogenesis and trehaloneogenesis [169]. Some monosaccharides contribute to the production of AA, and are feeding stimulants in insects (summarized in [139]). However, the utilization of carbohydrates depends on the ability of the insect species to hydrolyze polysaccharides. BSFL are able to use carbohydrate-based substrates and can convert glucose and xylose to lipids [170]. Best growth performance was observed using an optimized carbohydrate and protein ratio (21%:21%) and an optimized humidity (70%) of the substrate [26]. Nevertheless, compared to the dietary carbohydrate concentration, the protein concentration in the diet is of higher importance affecting fresh and dry body weight of the BSFL [171]. As shown by Barragan-Fonseca et al. the total protein and carbohydrate contents seem to be more important than the protein to carbohydrate ratio [148].

In mammals, glucose homeostasis is maintained by feedback mechanisms balancing glucose cellular import and replenishment to the blood. However, to our knowledge, the relationship between insulin signaling and glucose cellular import in BSF, unlike in *Drosophila melanogaster* [172], has not yet been clarified. Although there are similarities between insects regarding to insulin signaling and carbohydrate metabolism, fundamental differences between insects and vertebrates exist, e.g., that insects have trehalose as transport molecule for glucose in their hemolymph. As discussed by Shukla et al. this molecule exerts different further functions in insects, which are for example linked with the activation of chitin synthesis and stress recovery [173]. In holometabolous insects such as BSFL, along with lipid and glycogen deposits in the fat body [174], glycogen reserves in the midgut are essential for maintaining metabolic activity throughout the life cycle and during metamorphosis [106].

### Minerals

The inorganic component of the diet comprises minerals that can be analyzed in the ash fraction after combustion [135]. Depending on the rearing substrate, ash content of BSFL ranges from 5.1% to 15.8% of DM [41, 175, 176], which is higher than in several other farmed insect species [176, 177]. Mineral requirements of BSFL is largely unknown. This could be partly due to the relatively short history of this insect species to be used as a mini-livestock, but also due to the above-mentioned challenges in determining nutrient requirements of insects (see section Intestinal biology). For the conventional, vertebrate farmed animals, essential minerals in the diet are usually classified as macro- or major- and micro- or trace-minerals in order to address their requirement levels at g/d or % and at mg/d, respectively, per individual animal [135, 178]. This classification is unlikely to hold valid for insects, not only because of their small size and respective individual mineral requirements but also because of the differences in the functions of the minerals in vertebrates and invertebrates, that greatly vary in insects. In broad terms, minerals perform four main functions in vertebrate animals, which can be categorically classified as structural, physiological, catalytic and regulatory functions [179]. In vertebrate livestock mineral requirements are relatively well known (e.g. [179]). Minerals are required for the formation of the skeleton and other structural tissues (e.g. teeth), are used in the body in various compounds with particular functions, act as cofactors of enzymes, and are essential for maintaining the osmotic balance in the organism [178, 179]. In contrast, in most insects the cuticle, the main component of the exoskeleton, is a structure consisting mainly of chitin in a matrix with proteins, lipids and other compounds [180]. While the cuticle of BSFL and pupae of the face fly (*Musca autumnalis*) contain significant amounts of Ca, mineral content of cuticle in most insect species is insignificant [180, 181].

According to Thompson and Simpson [182], a complex mix of mineral ions is essential for insects. More specifically, insects require several metal ions that function as co-factor, and are included in metallo-enzymes. For instance, catalase includes iron, and copper is included in cytochrome oxidase, and in phenoloxidase. Insects additionally require Na, K and Cl [181]. Nevertheless, the balance of required minerals for insects is considerably different from that of mammals [182], and according to Chapman [181] commercial salt mixtures designed for vertebrates are not suitable for insects. Unlike vertebrates, most insects indeed require greater proportions of K, Mg, and P relative to Na, Ca and Cl [182]. Sorted by frequency, K, Ca, P, Mg, Na are the 5 most abundant minerals in the body of BSFL [41, 175, 176, 183]. In contrast to most farmed insect species, BSFL contain much higher amount of Ca than P. Average Ca:P ratio in several insect species ranges approximately from 1:4 to 1:17 [177], while the range of average Ca:P for BSFL is 1.2:1 to 8.2:1 [41, 175, 176, 183]. The higher Ca content and Ca:P ratio in the BSFL in comparison to other insect species is related to the chemical composition of the exoskeleton. Although the exoskeleton of most insect species is mainly composed of protein and chitin, BSFL have a so-called mineralized exoskeleton which explains the high calcium content [176]. More than a century ago, Johannsen [184] reported that Ca is deposited as calcium carbonate in the exoskeleton matrix of Stratiomyidae, an insect family to which BSF also belongs. Later on, Liland et al. demonstrated a link between dietary Ca level and Ca accumulation in BSFL, which is thought to be associated with its role in pupation of BSF [41].

It is generally accepted that investigations on mineral nutrition and metabolism are complicated by the way the functions of the various elements and other feed components interact with each other [135]. For example, the developmental rate of some insect species was reported to be proportional to the percentage of phosphorus in the diet [185], which holds also true for BSFL [59]. However, whether dietary P level has a causal effect on growth has not been fully elucidated. This is likely due to the potentially interacting or confounding effects of different dietary factors co-existing in the same diet, making it difficult to isolate the effects of a single dietary factor. For instance, as shown by Oonincx et al. [59] high protein diets often contain higher amounts of P than low protein diets, and BSFL fed on high protein/P diets grow better than those BSFL fed on low protein/P diets. The resulting higher growth is however neither attributable to protein nor P alone. By gradually increasing the amount of seaweed in the BSFL diet, Liland et al. produced diets with linearly decreasing protein and P levels that also varied in several other nutrients and energy concentrations [41]. Similar to the results of Oonincx et al. [59], Liland et al. [41] also observed improved BSFL growth parameters in response to increasing protein and P levels in the same diet.

Chapman et al. [181] addressed another challenge in identifying insect dietary requirements for micronutrients, including vitamins and minerals, that may not become apparent until at least two generations have passed since the maternal supply via eggs might meet nutritional requirement for one generation. Unlike micronutrients, insects’ requirements for macronutrients are expected to become apparent within a few days and certainly within a single generation [181]. As shown by Schmitt et al. BSFL accumulate several elements, including heavy metals and minerals (e.g. Cd, Hg, Pb, Ca, Mg, Mn and K) in the body with a bioaccumulation factor (BAF, i.e., mineral concentration found in BSFL relative to that of feeding substrate on DM basis) ranging from BAF > 1 to 9.1 [175]. The accumulation of heavy metals in the larvae constitutes a safety issue when the BSFL is to be used as feed for farmed animals. Using data provided by Shumo et al. [183] confirmed results of Schmitt et al. [175] that several minerals (e.g. K, Mg, Mn, Co) are indeed capable of accumulating in the BSFL irrespective of the feeding substrate of the larvae, whereas bioaccumulation of P, Ca, Na, Fe in the larvae show a dependency on the mineral content of the feeding substrate. However, these observations on BAF do not seem to be generally valid, as there seems to be a strong influence by the substrate in question [18]. Nonetheless, knowledge of BAF together with growth performance of BSFL could provide initial clues as to which minerals might be essential or toxic to BSFL. Concluding, mineral requirements of BSFL are largely unknown. More importantly, possible differences in mineral functions between vertebrates and invertebrates make the knowledge of mineral requirement developed for conventional farm animals not applicable to insect mini-livestock. Thus species-specific requirements of individual minerals need to be determined for insects, too.

## Conclusion and research needs

Compared to conventional monogastric livestock, BSFL show outstanding growth parameters and protein conversion ratio equivalent or better than in broiler and fish. However, feed and energy conversion ratios are poorer than in broiler and fish, reflecting the low performance with low-grade organic biomass or waste. Despite the growing interest in using BSFL products as components in livestock feed, our knowledge on the nutritional and physiological aspects of this insect species especially compared to conventional farmed animal species is scarce. The unique characteristics of BSFL compared to other livestock species are related to the function of their gastrointestinal tract, which enables larvae to efficiently utilize a wide range of substrates. Nevertheless, protein content, digestibility and amino acid composition of the feed substrate have been identified as very important factors affecting BSFL performance. Although capable of degrading dietary fiber, low-quality, high-fiber feeding substrates reduce BSFL performance and sustainability. To realize their potential to produce high quality protein by closing nutrient cycles in agro-ecological systems, more knowledge is needed on how to intelligently mix biowaste of different quality with food and feed industry by-products. This requires more knowledge about the abilities of BSFL to utilize fibrous and non-fibrous carbohydrates, the optimal carbon to nitrogen and the amino acid ratio, as well as the basis of their mineral metabolism, including research on the accumulation of potentially harmful substances. In addition, further knowledge is needed on the effects of forage physical structure on BSFL performance.

## Supplementary Information

**Additional file 1:** Supplementary Materials 1.

**Additional file 2:** Supplementary Table 1.

**Additional file 3.**

## Data Availability

The datasets used to generate Table 2 and Fig. 2 is available in a repository (https://doi.org/10.5281/zenodo.5886206). All other material is from published literature referenced in the reference list.

## Abbreviations

AA: Amino acids
BSF: Black soldier fly
BW: Body weight
BSFL: Black soldier fly larvae
CO_2_: Carbon dioxide
d: Day
GECR: Gross energy conversion ratio
FA: Fatty acid
FCR: Feed conversion ratio
GIT: Gastrointestinal tract
O_2_: Oxygen
PCR: Protein conversion ratio
RGR: Relative growth rate
RQ: Respiratory quotient
SGR: Specific growth rate
SCFA: Short-chain fatty acids
