# Supplementary material for: Growth efficiency, intestinal biology, and nutrient utilization and requirements of black soldier fly (Hermetia illucens) larvae compared to monogastric livestock species: a review
Source: J Anim Sci Biotechnol. 2022 May 5;13:31. doi: 10.1186/s40104-022-00682-7 (PMC9069764; doi:10.1186/s40104-022-00682-7)
Supplement: Supplementary file 1 — Additional file 1: Supplementary Materials 1. [file 40104_2022_682_MOESM1_ESM.docx]

# **Supplementary Materials 1** Source of data and calculation of parameters presented in **Table 2**

# Mean fattening period, relative and specific growth rates, feed conversion ratio, crude protein and gross energy conversion ratio (fresh matter) of black soldier fly larvae vs. meat producing monogastric livestock

## ***Paper selection and data collection***

To collect the relevant publications on performance related parameters of BSFL, chicken, pig and fish, three online databases were used including Pubmed, Scopus and Web of Science. The studies used to derive BSFL data include a wide range of feed sources such as chicken feed, fecal sludge, manure, kitchen waste, fruits and vegetables waste, fish, restaurant waste, by-products, dog food, abattoir waste, abattoir-fruit mix and vegetable waste and human feces mix. Feed conversion ratio (FCR) data for broilers and pigs were mostly extracted from meta-analysis studies and especially from studies that include different strains. For BSFL, body weight of the larvae one day after hatching was considered as the initial weight, and prepupae weight as the final weight. Pig birth weight and slaughter weight data were selected as they represent commercial conditions and procedures representative of the United States and Europe.

Initial larval weight (hatched weight) of fish (Atlantic salmon) was taken from Krejszeff et al. [1], and Nathanailides et al. [2]. Initial larvae (Post-Smolt) and market weight of two North American strains of Atlantic salmon; St. John River (SJR) salmon and Cascade salmon were derived from Davidson at el. [3]. Harvest weight of Scottish salmon between 2007 and 2017 was taken from Munro and Wallace [4]. Leclercq et al. [5] provided the market weight of sea-reared Atlantic salmon.

## ***Definition and calculations***

If not explicitly reported, feed intake during the whole raising period was back-calculated based on the FCR / body weight gain. Body weight gain (BWG) was calculated as market body weight – initial body weight. In addition, BWG was calculated based on dry matter. Dry matter content of whole body was taken from literature for BSFL [6-8], broilers [9-11], fish [12-14] and pig [15, 16].

Dry matter intake during the whole raising period was calculated as feed intake in the whole raising period × dry matter content of feed for different species. Dry matter content of feed and feed conversion ratio (g fresh matter feed / g body weight gain) for different species were based on literature for BSFL [6, 7, 17-21], broiler [22-25], fish [12, 26-28] and pig [29-31]. In addition, FCR based on feed intake /BWG DM and DMI/ BWG DM were calculated.

Protein conversion ratio was calculated as protein intake (g) / body weight gain (g) × 100; and gross energy conversion ratio as gross energy intake (MJ) / body weight gain (g) × 100.

Gross energy (GE) content of BSFL substrates and salmon fish was obtained from literature (for BSFL; [32-35] and for salmon fish: [12, 36, 37]). For pig and broilers ME content of the diet was obtained from the literature (for pig: [38-41], and for broiler; [24, 42-45]). Broiler metabolizable energy was converted to GE using the conversion factor of 1.379 [46]. For pigs, a conversion factor of 1.290 was used to convert ME to GE [47].

## ***Data availability through a repository***

All calculations and raw data used to generate Table 2 and Fig. 2 have been deposited in a repository (DOI:xxxxxxxxxxxxx to be done).

**Contents of the worksheets in the excel file:**

**1-Summary_all results**: Summary of all calculations regarding the growth and efficiency of different animal species. All calculations are traceable through formulas within and between different worksheets.

**2-BW_summary:** Summary of calculations made for average initial and market body weight as well as for raising period of different animal species. Data and calculations are given in the next worksheet (#3).

**3-BW_data**: this worksheet includes raw literature data for initial and market body weight as well as raising for period of different animal species. Wherever possible, n numbers used for the literature data were also presented here.

**4-FCR_summary**: This worksheet contains a summary of minimum, maximum and average FCR of different animal species calculated from the raw literature data.

**5-FCR_data:** Raw literature data used for calculation of FCR of different animal species.

**6-Substrate CP and GE_summary**: Summary of average values for gross energy Mj/kg in DM) and protein (% in DM) contents of the diets for different animal species.

**7-Substrate CP and GE_data**: Raw literature data for energy and protein contents of the diets for different animal species.

**8-Substrate DM summary data**: Average dry matter contents of the diets for different animal species estimated based on raw literature data

**9-Whole body DM summary+data**: Average dry matter content in whole body of different animal species using raw literature data

**10- References**: All references used for data collection and calculations are provided in this worksheet.

## ***References***

1. Krejszeff S, Żarski D, Palińska-Żarska K, Trąbska I, Kupren K, Targońska K, et al. Procedure for harmless estimation of fish larvae weight. Ital J Anim Sci. 2013;12(2):e44. DOI: <https://doi.org/10.4081/ijas.2013.e44>.

2. Nathanailides C, Stickland N, Lopez-Albors O. Influence of prehatch temperature on the development of muscle cellularity in posthatch Atlantic salmon (Salmo salar). Can J Fish Aquat Sci. 1995;52(4):675-80. DOI: <https://doi.org/10.1139/f95-068>.

3. Davidson J, May T, Good C, Waldrop T, Kenney B, Terjesen BF, et al. Production of market-size North American strain Atlantic salmon Salmo salar in a land-based recirculation aquaculture system using freshwater. Aquac Eng. 2016;74:1-16. DOI: <https://doi.org/10.1016/j.aquaeng.2016.04.007>.

4. Munro L, Wallace I. Marine Scotland Science—Scottish shellfish farm production survey 2016. Aberdeen: Marine Scotland Science; 2016.

5. Leclercq E, Taylor JF, Hunter D, Migaud H. Body size dimorphism of sea-reared Atlantic salmon (Salmo salar L.): implications for the management of sexual maturation and harvest quality. Aquaculture. 2010;301(1-4):47-56. DOI: <https://doi.org/10.1016/j.aquaculture.2010.01.029>.

6. Liland NS, Biancarosa I, Araujo P, Biemans D, Bruckner CG, Waagbø R, et al. Modulation of nutrient composition of black soldier fly (Hermetia illucens) larvae by feeding seaweed-enriched media. PloS One. 2017;12(8):e0183188. DOI: <https://doi.org/10.1371/journal.pone.0183188>.

7. Oonincx DG, Van Broekhoven S, Van Huis A, van Loon JJ. Feed conversion, survival and development, and composition of four insect species on diets composed of food by-products. PloS One. 2015;10(12):e0144601. DOI: <https://doi.org/10.1371/journal.pone.0144601>.

8. Van der Fels-Klerx H, Camenzuli L, Van Der Lee M, Oonincx D. Uptake of cadmium, lead and arsenic by Tenebrio molitor and Hermetia illucens from contaminated substrates. PLoS One. 2016;11(11):e0166186. DOI: <https://doi.org/10.1371/journal.pone.0166186>.

9. Bregendahl K, Sell J, Zimmerman D. Prediction of whole-body composition from the whole-body dry matter percentage of three-week-old broiler chicks. Poult Sci. 2002;81(8):1168-71. DOI: <https://doi.org/10.1093/ps/81.8.1168>.

10. Caldas JV, Boonsinchai N, Wang J, England JA, Coon CN. The dynamics of body composition and body energy content in broilers. Poult Sci. 2019;98(2):866-77. DOI: <https://doi.org/10.3382/ps/pey422>.

11. Strakova E, Suchý P, Navratil P, Karel T, Herzig I. Comparison of the content of crude protein and amino acids in the whole bodies of cocks and hens of Ross 308 and Cobb 500 hybrids at the end of fattening. Czech J Anim Sci. 2015;10(9.2):8-9. DOI: <https://10.17221/7976-CJAS>.

12. Aas TS, Ytrestøyl T, Åsgård T. Utilization of feed resources in the production of Atlantic salmon (Salmo salar) in Norway: An update for 2016. Aquac Rep. 2019;15:100216. DOI: <https://doi.org/10.1016/j.aqrep.2019.100216>.

13. Einen O, Roem A. Dietary protein/energy ratios for Atlantic salmon in relation to fish size: growth, feed utilization and slaughter quality. Aquac Nutr. 1997;3(2):115-26. DOI: <https://doi.org/10.1046/j.1365-2095.1997.00084.x>.

14. Hemre GI, Sagstad A, Bakke‐Mckellep A, Danieli A, Acierno R, Maffia M, et al. Nutritional, physiological, and histological responses in Atlantic salmon, Salmo salar L. fed diets with genetically modified maize. Aquac Nutr. 2007;13(3):186-99. DOI: <https://doi.org/10.1111/j.1365-2095.2007.00465.x>.

15. Smits C, Moughan P, Smith W. Chemical whole-body composition of the 20 kg liveweight growing pig. New Zealand J Agric Res. 1988;31(2):155-7. DOI: <https://doi.org/10.1080/00288233.1988.10417940>.

16. Lewis AJ, Southern LL. Swine nutrition: CRC press; 2000.

17. Sommer S, Hjorth M, Leahy J, Zhu K, Christel W, Sørensen C. Pig slurry characteristics, nutrient balance and biogas production as affected by separation and acidification. J Agric Sci. 2015;153(1):177-91. DOI: <https://doi.org/10.1017/S0021859614000367>.

18. Nicholson F, Chambers B, Smith K. Nutrient composition of poultry manures in England and Wales. Bioresour Technol. 1996;58(3):279-84. DOI: <https://doi.org/10.1016/S0960-8524(97)86087-7>.

19. Assessment of alternative phosphorus fertilizers for organic farming: sewage precipitation products [Internet]. 2015. Available from: <https://www.fibl.org/en/shop-en/article/c/fertilization/p/1665-sewage-precipitation-products.html>.

20. Retnani Y, Syananta F, Herawati L, Widiarti W, Saenab A. Physical characteristic and palatability of market vegetable waste wafer for sheep. Anim Prod. 2010;12(1).

21. Nguyen TT, Tomberlin JK, Vanlaerhoven S. Influence of resources on Hermetia illucens (Diptera: Stratiomyidae) larval development. J Med Entomol. 2013;50(4):898-906. DOI: <https://doi.org/10.1603/ME12260>.

22. Abdollahi M, Zaefarian F, Hall L, Jendza J. Feed acidification and steam-conditioning temperature influence nutrient utilization in broiler chickens fed wheat-based diets. Poult Sci. 2020;99(10):5037-46. DOI: <https://doi.org/10.1016/j.psj.2020.06.056>.

23. Berrocoso J, García-Ruiz A, Page G, Jaworski N. The effect of added oat hulls or sugar beet pulp to diets containing rapidly or slowly digestible protein sources on broiler growth performance from 0 to 36 days of age. Poult Sci. 2020;99(12):6859-66. DOI: <https://doi.org/10.1016/j.psj.2020.09.004>.

24. Brugaletta G, Luise D, De Cesare A, Zampiga M, Laghi L, Trevisi P, et al. Insights into the mode of action of tannin-based feed additives in broiler chickens: looking for connections with the plasma metabolome and caecal microbiota. Ital J Anim Sci. 2020;19(1):1349-62. DOI: <https://doi.org/10.1080/1828051X.2020.1842813>.

25. Lu J, Li Y, Qu L, Ma M, Yang X, Shen M, et al. Effects of energy-restricted feeding during rearing on sexual maturation and reproductive performance of Rugao layer breeders. Poult Sci. 2021:101225. DOI: <https://doi.org/10.1016/j.psj.2021.101225>.

26. Belghit I, Waagbø R, Lock EJ, Liland NS. Insect‐based diets high in lauric acid reduce liver lipids in freshwater Atlantic salmon. Aquac Nutr. 2019;25(2):343-57. DOI: <https://doi.org/10.1111/anu.12860>.

27. Bou M, Berge GM, Baeverfjord G, Sigholt T, Østbye T-K, Ruyter B. Low levels of very-long-chain n-3 PUFA in Atlantic salmon (Salmo salar) diet reduce fish robustness under challenging conditions in sea cages. J Nutr Sci. 2017;6. DOI: <https://doi.org/10.1017/jns.2017.28>.

28. Larsson T, Koppang EO, Espe M, Terjesen BF, Krasnov A, Moreno HM, et al. Fillet quality and health of Atlantic salmon (Salmo salar L.) fed a diet supplemented with glutamate. Aquaculture. 2014;426:288-95. DOI: <https://doi.org/10.1016/j.aquaculture.2014.01.034>.

29. Helm ET, Ross JW, Patience JF, Lonergan SM, Huff-Lonergan E, Greiner LL, et al. Nutritional approaches to slow late finishing pig growth: implications on carcass composition and pork quality. J Anim Sci. 2021;99(1):skaa368. DOI: <https://doi.org/10.1093/jas/skaa368>.

30. Chałabis-Mazurek A, Valverde Piedra JL, Muszyński S, Tomaszewska E, Szymańczyk S, Kowalik S, et al. The Concentration of Selected Heavy Metals in Muscles, Liver and Kidneys of Pigs Fed Standard Diets and Diets Containing 60% of New Rye Varieties. Animals. 2021;11(5):1377. DOI: <https://doi.org/10.3390/ani11051377>.

31. Ferrer P, García-Rebollar P, Calvet S, de Blas C, Piquer O, Rodríguez CA, et al. Effects of Orange Pulp Conservation Methods (Dehydrated or Ensiled Sun-Dried) on the Nutritional Value for Finishing Pigs and Implications on Potential Gaseous Emissions from Slurry. Animals. 2021;11(2):387. DOI: <https://doi.org/10.3390/ani11020387>.

32. Ulloa J, Van Weerd J, Huisman E, Verreth J. Tropical agricultural residues and their potential uses in fish feeds: the Costa Rican situation. Waste Manag. 2004;24(1):87-97. DOI: <https://doi.org/10.1016/j.wasman.2003.09.003>.

33. Danieli PP, Lussiana C, Gasco L, Amici A, Ronchi B. The effects of diet formulation on the yield, proximate composition, and fatty acid profile of the black soldier fly (Hermetia illucens L.) prepupae intended for animal feed. Animals. 2019;9(4):178. DOI: <https://doi.org/10.3390/ani9040178>.

34. Diem K, Lentner C. Wissenschaftliche Tabellen (7 ed.). CibaGeigy AG, Basel. Switzerland. 1968.

35. ALP. Eidgenössische Futtermitteldatenbank [Federal database of animal feed]: Forschungsanstalt Agroscope Liebefeld-Posieux ALP. Switzerland. 2007

36. Kaushik S, Gouillou-Coustans M, Cho C. Application of the recommendations on vitamin requirements of finfish by NRC (1993) to salmonids and sea bass using practical and purified diets. Aquaculture. 1998;161(1-4):463-74. DOI: <https://doi.org/10.1016/S0044-8486(97)00293-7>.

37. Ytrestøyl T, Aas TS, Åsgård T. Utilisation of feed resources in production of Atlantic salmon (Salmo salar) in Norway. Aquaculture. 2015;448:365-74. DOI: <https://doi.org/10.1016/j.aquaculture.2015.06.023>.

38. Kotrotsios N, Christaki E, Bonos E, Florou-Paneri P. Dietary carob pods on growth performance and meat quality of fattening pigs. Asian-Australas J Anim Sci. 2012;25(6):880. DOI: <https://10.5713/ajas.2011.11521>.

39. Kallabis KE, Kaufmann O. Effect of a high-fibre diet on the feeding behaviour of fattening pigs. Arch Anim Breed. 2012;55(3):272-84. DOI: <https://doi.org/10.5194/aab-55-272-2012>.

40. Szabó C, Jansman AJ, Babinszky L, Verstegen MW. The effect of high dietary fermentable carbohydrate content on the fattening performance and chemical body composition of fattening pigs. Poljoprivreda. 2007;13(1):55-60.

41. Yang P, Fan Y, Zhu M, Yang Y, Ma Y. Energy content, nutrient digestibility coefficient, growth performance and serum parameters of pigs fed diets containing tomato pomace. J Appl Anim Res. 2018;46(1):1483-9. DOI: <https://doi.org/10.1080/09712119.2018.1546181>.

42. Bozkurt M, Kucukyilmaz K, Çatli A, Çinar M. Growth performance and slaughter characteristics of broiler chickens fed with antibiotic, mannan oligosaccharide and dextran oligosaccharide supplemented diets. Int J Poult Sci. 2008.

43. Infante-Rodríguez F, Salinas-Chavira J, Montaño-Gómez M, Manríquez-Nuñez O, González-Vizcarra V, Guevara-Florentino O, et al. Effect of diets with different energy concentrations on growth performance, carcass characteristics and meat chemical composition of broiler chickens in dry tropics. Springerplus. 2016;5(1):1-6. DOI: <https://doi.org/10.1186/s40064-016-3608-0>.

44. Kamalzadeh A, Hosseini A, Moradi S. Effects of yeast glucomannan on performance of broiler chickens. Int J Agric Biol. 2009;11(1):49-53.

45. Abudabos AM. Optimal dietary phosphorus for broiler performance, bone integrity and reduction of phosphorus excretion. Asian J Anim Vet Adv. 2012;7:288-98. DOI: <https://10.3923/ajava.2012.288.298>.

46. National Research Council (NRC). Nutrient Requirements of Poultry: ninth revised edition1994.

47. Kil DY, Kim B, Stein H. Feed energy evaluation for growing pigs. Asian-Australas J Anim Sci. 2013;26(9):1205. DOI: <http://dx.doi.org/10.5713/ajas.2013.r.02>.
