# Supplementary material for: Growth efficiency, intestinal biology, and nutrient utilization and requirements of black soldier fly (Hermetia illucens) larvae compared to monogastric livestock species: a review
Source: J Anim Sci Biotechnol. 2022 May 5;13:31. doi: 10.1186/s40104-022-00682-7 (PMC9069764; doi:10.1186/s40104-022-00682-7)
Supplement: Supplementary file 2 — Additional file 2: Supplementary Table 1. [file 40104_2022_682_MOESM2_ESM.docx]

**Supplementary Table 1** Literature data of CO_2_ production and O_2_ consumption of selected insect species in different developmental stages

| Species | Develop-mental stage | Gas exchange measurement | Specified condition | Temperature, °C | Fed during measurement | CO_2_ production  µl/mg BW/h | O_2_ consumption,  µl / mg BW/h | Reference |
| --- | --- | --- | --- | --- | --- | --- | --- | --- |
| *Acheta domesticus* | 5^th^, 6^th^ instar nymph | Continuous^1^ | Activity^5^ | 28 | Yes | 38.50 | - | [1] |
| *Blaptica dubia* | Nymphs | Continuous | Activity^5^ | 28 | Yes | 10.8 | - | [1] |
| *Drosophila melanogaster* | Imago | Discontinuous^2^ | Resting | - | No | 2.8 | - | [2] |
| *Drosophyla melanogaster* | Larva | Continuous | Activity^6^ | 25 | No | 1.7 | 2.1 | [3] |
| *Drosophila melanogaster*^4^ | Imago | Continuous | Resting^7^ | - | No | 4.9^3^ | - | [4] |
| *Drosophila melanogaster* | Imago | Continuous | Resting | 20 | No | 0.3 | - | [5] |
| *Hermetia illucens* | Larvae | Discontinuous | Activity^8^ | 28 | Yes | 49.8 |  | [6, 7] |
| *Forelius mccooki* | Imago | Continuous | Resting | 20 | No | 1.9 | - | [5] |
| *Locusta migratoria* | 3^rd^, 4^th^ instar nymphs | Continuous | Activity^5^ | 32 | Yes | 63.1 | - | [1] |
| *Manduca sexta* | Larva, 5^th^instar | Discontinuous | Resting^9^ | 25 | No | - | 2.1^3^ | [8] |
| *Pachnoda Marginata* | Larva, 3^rd^instar | Continuous | Activity^5^ | 28 | Yes | 28.3 | - | [1] |
| *Pogonomyremex californicus* | Imago | Discontinuous | Resting | 20 | No | 1.7 | - | [5] |
| *Tenobrio molitor* | Larva, 5^th^instar | Continuous | Activity^5^ | 25 | Yes | 34.9 | - | [1] |

^1^Continuous = flow-through system for gas exchange measurement with data points of high density.

^2^Discontinuous = stop-flow system for gas exchange measurement; only one or few data points available.

^3^Data taken from a graph.

^4^Mean value of female and male flies.

^5^Moving activity within containers with adequate feed.

^6^Moving activity within a 5 ml syringe, feed deprived condition.

^7^Resting; method according to Van Voorhies et al. 2004, with modifications.

^8^Activity; larvae within compost substrate.

^9^Resting or restricted mobility within a test tube.

**References**

1. Oonincx DG, van Itterbeeck J, Heetkamp MJ, van den Brand H, van Loon JJ, van Huis A. An exploration on greenhouse gas and ammonia production by insect species suitable for animal or human consumption. PLoS One. 2010;5(12):e14445. DOI: 10.1371/journal.pone.0014445.

2. Van Voorhies WA, Khazaeli AA, Curtsinger JW. Testing the "rate of living" model: further evidence that longevity and metabolic rate are not inversely correlated in Drosophila melanogaster. J Appl Physiol (1985). 2004;97(5):1915-22. DOI: 10.1152/japplphysiol.00505.2004.

3. Callier V, Hand SC, Campbell JB, Biddulph T, Harrison JF. Developmental changes in hypoxic exposure and responses to anoxia in Drosophila melanogaster. J Exp Biol. 2015;218(Pt 18):2927-34. DOI: 10.1242/jeb.125849.

4. Jumbo-Lucioni P, Ayroles JF, Chambers MM, Jordan KW, Leips J, Mackay TF, et al. Systems genetics analysis of body weight and energy metabolism traits in Drosophila melanogaster. BMC Genomics. 2010;11:297. DOI: 10.1186/1471-2164-11-297.

5. Lighton JR, Schilman PE, Holway DA. The hyperoxic switch: assessing respiratory water loss rates in tracheate arthropods with continuous gas exchange. J Exp Biol. 2004;207(Pt 25):4463-71. DOI: 10.1242/jeb.01284.

6. Ermolaev E, Lalander C, Vinneras B. Greenhouse gas emissions from small-scale fly larvae composting with Hermetia illucens. Waste Manag. 2019;96:65-74. DOI: 10.1016/j.wasman.2019.07.011.

7. Ermolaev E, Lalander C, Vinneras B. Greenhouse gas emissions from small-scale fly larvae composting with Hermetia illucens. Waste Manage. 2019;96:65-74. DOI: 10.1016/j.wasman.2019.07.011.

8. Callier V, Nijhout HF. Control of body size by oxygen supply reveals size-dependent and size-independent mechanisms of molting and metamorphosis. Proc Natl Acad Sci U S A. 2011;108(35):14664-9. DOI: 10.1073/pnas.1106556108.
